# Exploring the Effect and Mechanism of Si-Miao-Yong-An Decoction on Abdominal Aortic Aneurysm Based on Mice Experiment and Bioinformatics Analysis

**DOI:** 10.1155/2022/4766987

**Published:** 2022-05-31

**Authors:** Zhenyu Xu, Lulu Zhang, Ning Huangfu, Fengchun Jiang, Kangting Ji, Shenghuang Wang

**Affiliations:** ^1^Department of Cardiology, Ningbo First Hospital, Ningbo, Zhejiang, China; ^2^Shanghai Medical College of Fudan University, Shanghai, China; ^3^Department of Cardiology, Hangzhou Red Cross Hospital, Hangzhou, Zhejiang, China; ^4^Department of Cardiology, Second Affiliated Hospital & Yuying Children's Hospital of Wenzhou Medical University, Wenzhou, Zhejiang, China

## Abstract

**Background:**

Abdominal aortic aneurysm (AAA) is a fatal disease characterized by high morbidity and mortality in old population. Globally, effective drugs for AAA are still limited. Si-Miao-Yong-An decoction (SMYAD), a traditional Chinese medicine (TCM) formula with a high medical value, was reported to be successfully used in an old AAA patient. Thus, we reason that SMYAD may serve as a potential anti-AAA regime.

**Objective:**

The exact effects and detailed mechanisms of SMYAD on AAA were explored by using the experimental study and bioinformatics analysis.

**Methods:**

Firstly, C57BL/6N mice induced by Bap and Ang II were utilized to reproduce the AAA model, and the effects of SMYAD were systematically assessed according to histology, immunohistochemistry, and enzyme-linked immunosorbent assay (ELISA). Then, network pharmacology was applied to identify the biological processes, pathways, and hub targets of SMYAD against AAA; moreover, molecular docking was utilized to identify the binding ability and action targets.

**Results:**

In an animal experiment, SMYAD was found to effectively alleviate the degree of pathological expansion of abdominal aorta and reduce the incidence of Bap/Ang II-induced AAA, along with reducing the damage to elastic lamella, attenuating infiltration of macrophage, and lowering the circulating IL-6 level corresponding to the animal study, and network pharmacology revealed the detailed mechanisms of SMYAD on AAA that were related to pathways of inflammatory response, defense response, apoptotic, cell migration and adhesion, and reactive oxygen species metabolic process. Then, seven targets, IL-6, TNF, HSP90AA1, RELA, PTGS2, ESR1, and MMP9, were identified as hub targets of SMYAD against AAA. Furthermore, molecular docking verification revealed that the active compounds of SMYAD had good binding ability and clear binding site with core targets related to AAA formation.

**Conclusion:**

SMYAD can suppress AAA development through multicompound, multitarget, and multipathway, which provides a research direction for further study.

## 1. Introduction

Abdominal aortic aneurysm (AAA) is a life-threatening vascular disease, mainly associated with the risk factors that include male gender, smoking, and old age [1]. AAA develops gradually and imperceptibly, and it is the tenth-leading killer of men older than 55 years in the United States [2]. In clinic, AAA is defined as a maximum abdominal aortic diameter at least 1.5 times larger than the expected normal value [3]. For now, open or endovascular surgical repair is useless for most small AAA, and the specific medicine against AAA remains undeveloped [4]. Nowadays, AAA can be identified at early stage as a result of imaging and screening programs [5]. Thus, it is important to develop effective medical therapies that prevent the progressive expansion and rupture of AAA. For decades, large amount of research studies were performed to increase the understanding of AAA pathogenesis and pathological features, including inflammation responses, oxidative stress, smooth muscle apoptosis, and extracellular matrix (ECM) degeneration [6–10]. Previous studies suggested that statins may have a potential effect on AAA [11, 12]. But in fact, statins' clinical effect is not satisfactory, and it has some side effects such as causing severe liver damage [13, 14]. Therefore, the focus should now be on searching new drugs with better efficacy and fewer side effects.

According to the dialectical treatment of TCM, AAA was primarily related to the blood stasis. Si-Miao-Yong-An decoction (SMYAD) is a traditional Chinese medicine (TCM) that has been used safely for hundreds of years. This classic ancient recipe was traditionally used for gangrene [15] and in the modern medicine therapy system to treat peripheral vascular diseases [16]. Among the four herbs included in SMYAD, *Flos Lonicerae* (Chinese name Jin-Yin-Hua), as the principal drug, has the efficacy of clearing away heat and detoxifying; *Radix Scrophulariae* (Chinese name Xuan-Shen) and *Radix Angelica Sinensis* (Chinese name Dang-Gui) are both adept in invigorating blood circulation and eliminating blood stasis; and *Radix Glycyrrhizae* (Chinese name Gan-Cao), as the mediator drug, coordinates the drug actions. Consequently, SMYAD can be used to promote poor blood circulation and remove detoxify, which means that it has the corresponding theoretical basis of TCM in the treatment of AAA. Moreover, SMYAD has been attempted to use in an old AAA patient and the clinic effect was fine [17]. However, the pharmacological effects, related pathways, and therapeutic core targets of SMYAD for treating AAA are still not well understood, which limit its wide use in clinical practice and further development.

Chinese materia medica is a complicated system of multicompounds, multitargets, and synergistic effect among compounds. Articulating the relationship among these elements is a difficult but fascinating challenge. Encouragingly, bioinformatics analysis provides us an up-to-date perspective to understand the interactions of compounds, pathways, and targets with disease [18]. It can also help us to efficiently screen potential drugs and comprehensively evaluate therapeutic mechanisms and targets of drugs on diseases. In this article, histology, immunohistochemistry, and enzyme-linked immunosorbent assay (ELISA) were used to evaluate the exact effects of SMYAD on 3, 4-benzopyrene (Bap)/angiotensin II (Ang II)-induced AAA mice. Then, bioinformatics analysis, including network pharmacology and molecular docking, was further utilized to perform visual analysis on the interaction of SMYAD and AAA, to identify the mechanisms, core targets, the potent compounds of SMYAD, and their targets in the treatment of AAA. The above all results suggested that SMYAD may be a potentially effective drug for AAA.

## 2. Materials and Methods

### 2.1. Experimental Animal

Male C57BL/6N mice, 6-7 months old, weighing 28–32 g were supplied by Charles River (Beijing, China) and maintained on a 12:12-hour light-dark cycle with free ad libitum access to food and water for a one-week acclimatization period. Animal experiments were conducted in accordance with the guidelines for animal experiments of Wenzhou Medical University and approved by the animal ethics committee.

### 2.2. Preparation of SMYAD

Daily doses of SMYAD recorded in “*Yanfangxinpian*” (Qing dynasty) were *Flos Lonicerae* (90 g), *Radix Scrophulariae* (90 g), *Radix Angelica Sinensis* (60 g), and *Radix Glycyrrhizae* (30 g). The abovementioned raw herbs were all obtained from Tongrentang pharmacy. According to the procedures of automatic decocting machine (HYDY-5), two copies of daily raw herbs (270 g × 2) were washed thoroughly and put into the decocting pot. The raw herbs were soaked in distilled water (1.5 L × 2) for 1h and then decocted. Extracted solution (0.5 L × 2) was obtained after secondary filtered, and the concentration of the collected solution was equivalent to 0.54 g raw herbs/ml. The experimental dosage of SMYAD was determined according to the body surface conversion between human and mouse. Finally, the above solution was evaporated to the concentration of 13.2 g raw herbs/ml (defined as SMYAD-low dose) and 26.4 g raw herbs/ml (defined as SYMAD-high dose) by a rotary evaporator (LC-RE-5000). The above operations were repeated every day to prepare SMYAD.

### 2.3. Animal Grouping and Intervention

After one-week acclimatization, 42 mice were weighed and then randomly divided into two groups (count this day as Day 1): normal control group (*n* = 6, control for short) and model group (*n* = 36). From the Day 1 to Day 42, the 6 mice in control received no Bap/Ang II and other treatments; the 36 mice in the model group were injected intraperitoneally with Bap (B1760, Sigma-Aldrich) at a dose of 10 mg/kg body weight weekly and a dose (0.72 mg/kg/day) of Ang II (A9525, Sigma-Aldrich) using subcutaneous osmotic mini-pumps (Model# 2006, Alzet). Current guideline for use of bio-hazardous materials was followed when using Bap. At Day 15, 36 mice in the model group were randomly and equally divided into three groups, namely, Bap-/Ang II-treated + saline group (Bap + Ang II for short, *n* = 12), Bap-/Ang II-treated + SMYAD-low-dose group (Bap + Ang II + SL for short, *n* = 12), and Bap-/Ang II-treated + SMYAD-high-dose group (Bap + Ang II + SH for short, *n* = 12). From Day 15 to Day 42, mice in Bap + Ang II + SL and Bap + Ang II + SH were fed intragastrically 1 ml of SMYAD-low dose or SMYAD-high dose once daily, respectively; meanwhile, mice in Bap + Ang II were fed intragastrically with 1 ml of saline solution once daily. On Day 43, all survival mice were weighed and then euthanized.

### 2.4. Evaluation of Aortas and Histological Examination

After euthanasia, the thoracic and abdominal cavities were probed, and the aorta was sequentially irrigated with PBS and 4% formaldehyde through the left ventricle. Macroscopic examination of aortas was performed and carefully cleaned under a dissection microscope. The suprarenal segments or obvious lesions of abdominal aorta were dissociated and fixed in 10% paraformaldehyde and then embedded in paraffin wax. The paraffin slices of the abdominal aortic sections (4 *μ*m thick) were prepared and stained with haematoxylin-eosin (HE) stain. HE stain was used to assess the morphology and structure of abdominal aorta. Due to the irregular shapes of slices, the perimeter, instead of diameter, was measured with ImageJ for comparison. For each animal, two slices of abdominal aorta were selected and measured, and then, the average perimeter was calculated and recorded. The abdominal aorta with perimeter increases equaled or greater than 50% of average perimeter of mice in control was considered AAA formation. The hearts of mice were also collected and weighed.

### 2.5. Identification of Macrophage by Immunohistochemistry Staining

The paraffin slices of the abdominal aortic sections (4 *μ*m thick) were examined for macrophage infiltration by immunohistochemistry staining with the monoclonal CD68 antibody (MA5-16674, Invitrogen) against mouse macrophage according to the streptavidin-peroxidase (SP) method. Brown or dark brown staining in cytoplasm was defined as positive expression. The extensity of macrophage infiltration within the abdominal aorta was quantified with Image-Pro Plus (IPP) 6.0 software by the number of positively stained cells per 0.01 mm^2^. For each animal, two slices of stained abdominal aortic sections were selected and the average number of positive cells was recorded by an investigator who was unaware of sample's identity.

### 2.6. Measurement of Circulating Interleukin-6 (IL-6) Levels

The peripheral blood sample was collected from mouse tail at Day 1 and Day 43. After 20-minute centrifugation, serum samples were obtained and stored at −80°C until analysis. The circulating IL-6 levels were detected with Avidin-Biotin complex (ABC)-ELISA by commercially available ELISA kit (M6000B, R&D). Testing was performed independently by a researcher who was unaware of sample's identity.

### 2.7. Statistical Analysis

Exploratory data analysis and Shapiro–Wilk tests were performed to determine the normality of the data distribution. Continuous variables are expressed as mean ± SD unless otherwise noted. The self differences analyzed by paired-*t* test; differences among groups were analyzed by one-way ANOVA, followed by Dunnett's T3 test. For the incidence of AAA, counts and percentages are presented. Differences among groups were analyzed by Fisher's exact test, followed by the Bonferroni test. The relationship of two independent normality quantitative samples was analyzed by linear correlation. All analyses were conducted with statistical SPSS software 25.0 (IBM Corp., Armonk, NY, USA). The level of significant difference was set as a 2-sided *P* < 0.05.

### 2.8. Screening Active Compounds and Corresponding Targets

The compounds of SMYAD were screened out from the Traditional Chinese Medicine Systems Pharmacology Database and Analysis Platform (TCMSP) database (https://tcmsp-e.com/,Ver.2.3) [19]. Then, the assessment of absorption, distribution, metabolism, and excretion (ADME) was employed to select active compounds that contribute to its therapeutic effects, while those with bad drug ability compounds were removed. In order to obtain compounds with higher oral absorption and utilization, the active compounds were required to meet two of the following parameters: (1) oral bioavailability (OB) equal to or greater than 30% and (2) drug likeness (DL) equal to or greater than 0.18. The canonical smiles and PubChem ID of the above active compounds were calibrated using PubChem (https://pubchem.ncbi.nlm.nih.gov/) [20]. Then, the corresponding target proteins with the area under curve (AUC) equal to or greater than 0.75 and possibility equal to or greater than 0.50 were obtained from TargetNet (https://targetnet.scbdd.com) [21]. Finally, the target proteins of SMYAD reviewed in humans were transformed into gene symbols by the UniProt database (https://www.uniprot.org) [22].

### 2.9. Construction of Network of “Compounds-Targets”

Excel files containing the information of SMYAD-matched targets were established. Next, they were imported into Cytoscape 3.7.2 software [23]. Finally, the “compounds-targets” network was constructed and visualized by Cytoscape.

### 2.10. Collection of AAA-Related Targets

“Abdominal aortic aneurysm” and “aortic aneurysm” were selected and set as searching keywords, and then, AAA-related targets were respectively screened from four sources: (1) GeneCards database (https://www.genecards.org/) [24] with relevance score equal to or greater than 7.14; (2) CTD database (https://ctdbase.org/) [25] with inference score equal to or greater than 16.5; (3) DisGeNET database (https://www.disgenet.org/home/) [26] with a score equal to or greater than 0.02; and (4) OMIM database (https://www.omim.org/geneMap) [27]. Then, all AAA-related targets were amalgamated. Only “*Homo sapiens*” targets linked to AAA were selected and verified by their unique UniProtKB ID and target names in the UniProt database.

### 2.11. Screening the Common Targets of Drug and Disease

The SMYAD-matched targets and AAA-related targets were imported into the Bioinformatics platform (https://www.bioinformatics.com.cn/) to obtain a Venn diagram.

### 2.12. Enrichment Analysis

Two parts were included in enrichment analysis: Gene Ontology (GO) functional enrichment [28] and Kyoto Encyclopedia of Genes and Genomes (KEGG) pathway enrichment [29]. Then, the common targets of SMYAD and AAA were imported into the “Multiple Gene List” and implemented “Custom Analysis” in Metascape platform (https://metascape.org) [30]. The GO functional enrichment analysis includes biological process (BP), molecular function (MF), and cellular component (CC). Then, the enrichment analyses were carried out with criteria as Min.overlap = 3, *P* value cutoff = 0.01, and Min. enrichment = 1.5. The entries of GO were chosen and listed. The BP and KEGG pathways were selected out and visualized by Cytoscape.

### 2.13. Protein-Protein Interaction (PPI) Network Construction and Hub Target Screening

PPI network provides information regarding the predicted and experimental interactions of proteins. The STRING database (https://www.string-db.org) [31] was utilized to perform analysis with the condition of min required interaction score >0.40. Then, the obtained PPI network was visualized by Cytoscape. Finally, the hub targets in the network were identified and ranked according to Maximal Clique Centrality (MCC) by CytoHubba algorithms [32].

### 2.14. Molecular Docking Verification

The 3D chemical structural formulas of chosen compounds were obtained from PubChem and energy minimizing employed to ChemBioDraw 3D. The crystal structures of selected targets were collected from the protein data bank (PDB) (https://www.rcsb.org) [33] for molecular docking. According to the results, the bioinformatics platform was used to draw a heat map and clustering analysis. The platform used a green, black, and red tricolor in a 100-color mode. The platform sorted the rows and columns based on the hierarchical clustering result. Then, colors were assigned to represent the binding affinity. The image was processed and visualized with a condition of scale direction (*Z*-score)—row, clustering direction—bidirectional, clustering method—complete, and distance method—correlation. Moreover, the binding affinity, sites, and interactions between active compounds and potential targets were achieved and analyzed by classical molecular dynamics using Auto Dock Tools-1.5.6, Pymol 2.3, and Discovery Studio 4.5 Client.

## 3. Results

### 3.1. SMYAD Inhibited Bap/Ang II-Induced AAA Formation in Mice

**Figure 1:**
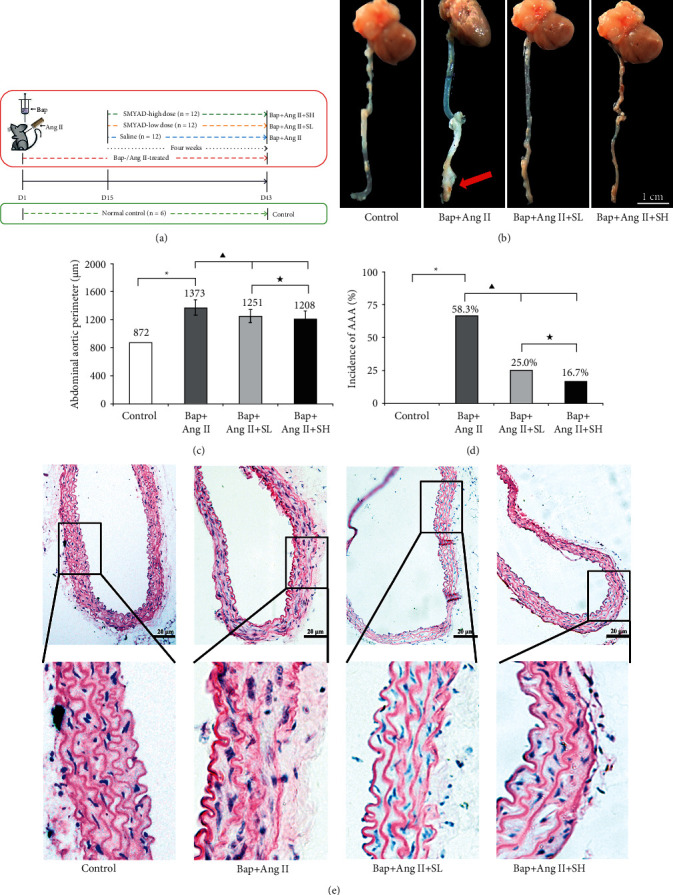
Si-Miao-Yong-An decoction (SMYAD) inhibited 3, 4-benzopyrene (Bap)/angiotensin II (Ang II)-induced AAA in mice. (a) Timeline of experimental period. (b) Representative images of abdominal aorta from normal control group (control for short), Bap-/Ang II-treated + saline group (Bap + Ang II for short), Bap-/Ang II-treated + SMYAD-low-dose group (Bap + Ang II + SL for short), and Bap-/Ang II-treated + SMYAD-high-dose group (Bap + Ang II + SH for short). No obvious pathologic expansion or hematoma formation was observed in the control. The arrow indicated pathologic expansion and abdominal aortic aneurysm (AAA) formation in Bap + Ang II. And the pathologic expansion of abdominal aortas in Bap-/Ang II-treated mice was both alleviated by SMYAD-low dose and SMYAD-high dose. (c) The perimeter of selected abdominal aortas (*μ*m) in control (*n* = 6), Bap + Ang II (*n* = 12), Bap + Ang II + SL (*n* = 12), and Bap + Ang II + SH (*n* = 12) was measured and recorded at Day 43. Data represented the ratio or mean ± SD.^*∗*^*P* < 0.05 vs control, ^▲^*P* < 0.05 vs Bap + Ang II, ^★^*P* > 0.05 vs Bap + Ang II + SH. (d) Incidences of AAA (%) in control (0/6), Bap + Ang II (8/12), Bap + Ang II + SL (3/12), Bap + Ang II + SH (2/12). ^*∗*^*P* < 0.05 vs control, ^▲^*P* < 0.05 vs Bap + Ang II, ^★^*P* > 0.05 vs Bap + Ang II + SH. ((e) ×20) Representative photomicrographs of abdominal aortic tissues were evaluated by using haematoxylin-eosin (HE) staining. Elastin lamella was continuous and complete in control. The elastic lamella lost fine lines and instead degraded into loosely defined lines in Bap + Ang II, and the severity of elastin lamella destruction was partly attenuated by receiving both SMYAD-low dose and SMYAD-high dose. Scale bar, 20 *μ*m.

The timeline of Bap-/Ang II-treated AAA model and SMYAD treatment is shown in Figure 1(a). During the experimental period, no mice died accidentally. Moreover, the excretion and behavior of mice received SMYAD-low dose and SMYAD-high dose were both normal. And the weights of mice in four groups were similar at Day 1 and Day 43 (Supplementary Materials Table S1). Abdominal aorta of each animal was isolated and examined for morphological evaluation. Representative photomicrographs of each group were shown in Figure 1(b). Quantifications of morphological changes of abdominal aortas in four groups are shown in Figures 1(c) and 1(d). The perimeter of abdominal aorta was significantly increased in Bap + Ang II (1373 ± 113 *μ*m) than that in control (872 ± 31 *μ*m), which indicated the successful establishment of the AAA model. The perimeter of abdominal aorta was significantly decreased, in both Bap + Ang II + SL (1251 ± 93 *μ*m) and Bap + Ang II + SH (1208 ± 114 *μ*m), compared to Bap + Ang II. Similarly, in comparison with control (0/6), the incidence of AAA was significantly increased to 66.7% (8/12) in Bap + Ang II (*P* < 0.05). The incidence of AAA was presented at 25.0% (3/12) and 16.7% (2/12) in Bap + Ang II + SL and Bap + Ang II + SH, respectively, which was significantly decreased compared to Bap + Ang II. Meanwhile, there was no significant difference in these indexes between Bap + Ang II + SL and Bap + Ang II + SH.

### 3.2. SMYAD Alleviated the Damage to Elastin Lamella Induced by Bap and Ang II

HE staining was used to evaluate the histological structure of abdominal aorta, especially for elastic lamella. Elastic lamella was known to closely related to the stability of vascular structure [34]. As shown in Figure 1(e), a representative image of HE-stained Bap + Ang II showed severe damage to vascular structure, disarray, and degradation of elastic lamella. Administrations of SMYAD could partly reverse the detrimental effects of Bap/Ang II on the elastin lamella, thereby inhibiting pathologic dilation of abdominal aorta and reducing the incidence of AAA. Furthermore, the continuity and integrity of elastin lamella in Bap + Ang II + SH was better than those in Bap + Ang II + SL.

### 3.3. SMYAD Mitigated Macrophage Infiltration into the Abdominal Aorta

**Figure 2:**
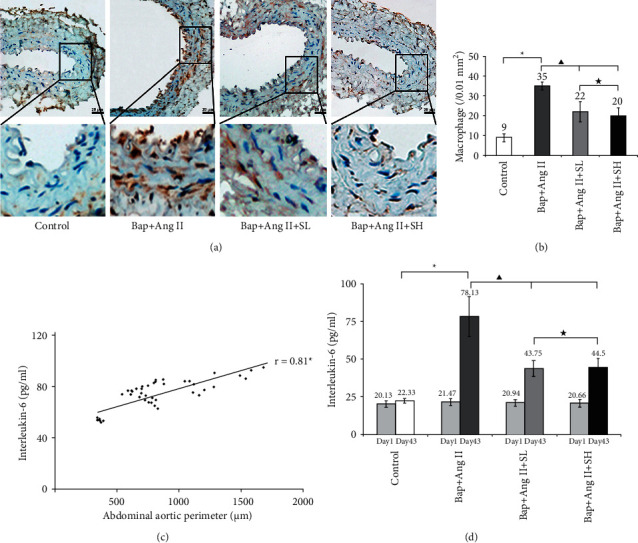
SMYAD inhibited Bap/Ang II-induced inflammatory response in mice. ((a) ×20) Representative photomicrographs of immunohistochemistry staining with macrophage CD68 antibody of abdominal aortic sections from four groups. Brown or dark brown staining in cytoplasm was defined as positive expression. Scale bar 20 *μ*m. (b) Quantification of macrophage infiltration in abdominal aortic sections. Data represented the mean ± SD. ^*∗*^*P* < 0.05 vs control,^▲^*P* < 0.05 vs Bap + Ang II, ^★^*P* > 0.05 vs Bap + Ang II + SH. (c) There was a positive correlation between circulating interleukin-6 (IL-6) levels and abdominal aortic perimeters (*r* = 0.81, ^*∗*^*P* < 0.05). (d) Quantification of circulating IL-6 levels at Day 1 and Day 43. Data represented the mean ± SD.^*∗*^*P* < 0.05 vs control, ^▲^*P* < 0.05 vs Bap + Ang II, ^★^*P* > 0.05 vs Bap + Ang II + SH.

Vascular macrophage infiltration was a clear inflammatory response, which contributed to the development of AAA and was a key event in AAA. Macrophages within the abdominal aorta were detected by immunohistochemistry staining (Figure 2(a)). The extensity of macrophage infiltration was quantified by counting the positively stained cells per 0.01 mm^2^ (Figure 2(b)). The results showed the extensity of macrophage infiltration was significantly aggravated in Bap + Ang II (50 ± 5) compared to control (11 ± 2), which indicated that Bap/Ang II-induced AAA was accompanied by numerous infiltrations of macrophage. And the response was significantly inhibited by either SMYAD-low dose (23 ± 3) or SMYAD-high dose (21 ± 3). The extensity of macrophage infiltration in Bap + Ang II + SH was slighter than that in Bap + Ang II + SL, but the difference was not significant.

### 3.4. SMYAD Decreased Circulating Inflammation Mediator Levels

IL-6 was a clear and important mediator of inflammation, and increased circulating concentration of IL-6 had been found in patients with AAA [35]. Therefore, the circulating IL-6 levels were measured to observe the anti-inflammation effect of SMYAD on Bap-/Ang II-treated mice. As shown in Figure 2(c), there was a high positive correlation between circulating IL-6 levels and abdominal aortic perimeters (*r* = 0.81). The results shown in Figure 2(d) indicated that Bap/Ang II-induced AAA development was along with an elevated circulating IL-6 level. The circulating IL-6 level in Bap + Ang II (78.13 ± 13.13 pg/ml) was significantly increased compared to control (22.33 ± 1.58 pg/ml). And the circulating IL-6 level was significantly decreased, in both Bap + Ang II + SL (43.75 ± 5.29 pg/ml) and Bap + Ang II + SH (44.50 ± 5.93 pg/ml), compared to Bap + Ang II. There was no significant difference in circulating IL-6 levels between Bap + Ang II + SL and Bap + Ang II + SH.

### 3.5. Compound Screening, Target Prediction of SMYAD, and Network of “Compounds-Targets”

**Figure 3:**
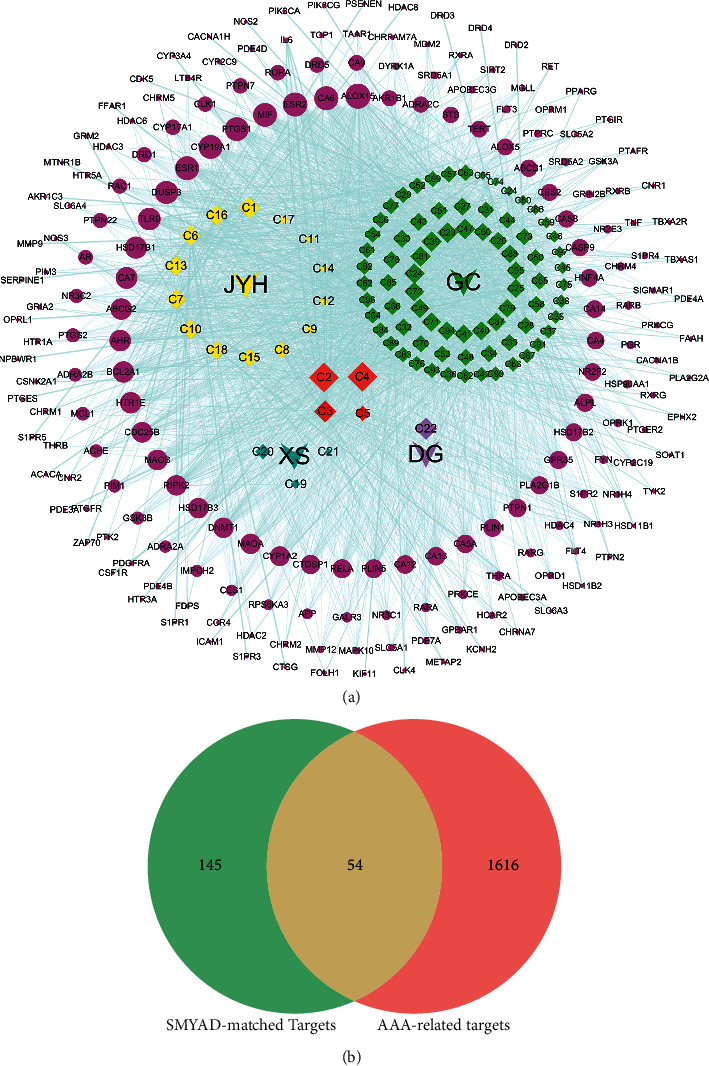
Compounds-target network and Venn diagram of SMYAD-matched and AAA-related targets of SMYAD and AAA. (a) The network of 97 active compounds from SMYAD and 199 SMYAD-matched targets (sorted by nodes degree). The rose-red ellipse nodes represented target genes. The diamond nodes represented compounds (yellow for *Flos Lonicerae*, JYH for short; blue for *Radix Scrophulariae*, XS for short; purple for *Radix Angelicae Sinensis*, DG for short; green for *Radix Glycyrrhizae*, GC for short). C2, C4 were the common compounds of JYH and GC; C3 was the common compound of JYH, XS, DG, and GC; C5 was the common compound of JYH and XS. Among all active compounds, C2, C4 were the highest degree value nodes. (b) The Venn diagram of 199 SMYAD-matched and 1670 AAA-related targets.

In order to determine SMYAD-associated targets, a series of bioinformatics analyses were conducted. As shown in Table 1 and Supplementary Materials Table S2, a total of 97 active compounds of SMYAD were assayed from TCMSP and SwissADME, including 18 in *Flos Lonicerae*, 4 in *Radix Scrophulariae*, 3 in *Radix Angelica Sinensis*, and 78 in *Radix Glycyrrhizae*. Among all compounds, quercetin (C2), beta-sitosterol (C3), kaempferol (C4), and stigmasterol (C5) were the common compounds. A total of 199 SMYAD-matched targets were identified and selected from TargetNet. In order to elucidate the inner relationship between the active compounds and SMYAD-matched targets clearly, the Cytoscape software was used to establish the “compounds-targets” network (Figure 3(a)).

### 3.6. AAA-Related Targets and Common Targets

A total of 1670 targets, identified from GeneCards, CTD, DisGeNET, and OMIM, were considered AAA-related targets. Then, SMYAD-matched targets and AAA-related targets were compared, 54 SMYAD-matched targets against AAA were identified (Figure 3(b)), and the common targets were subjected to further analysis.

### 3.7. Go and KEGG Pathway Enrichment Analysis

**Figure 4:**
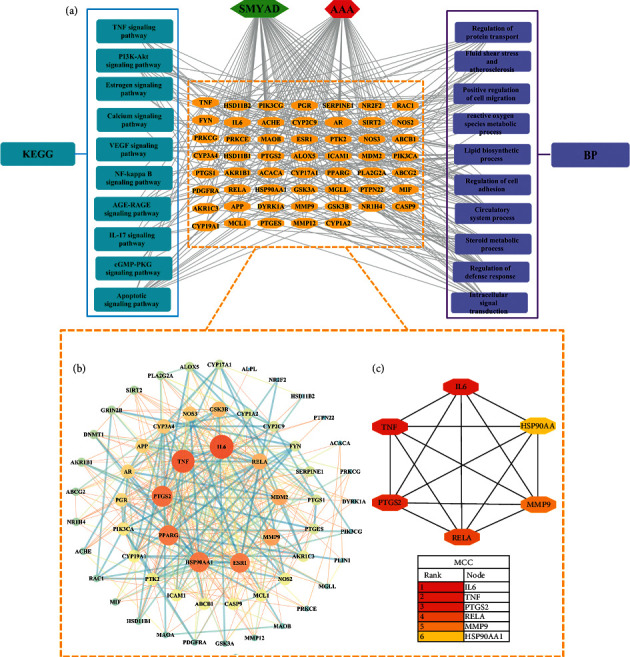
Network pharmacology-based elucidations of mechanisms of SMYAD on AAA. (a) Interaction network of SMYAD-target-biological process (BP)-Kyoto Encyclopedia of Genes and Genomes (KEGG)-AAA. The orange nodes represented the common targets of SYMAD and AAA, and the enriched BP and KEGG pathways related to inflammation and elastin lamella were selected out of top 20 *q*-value entries. (b) Protein-protein interaction (PPI) network was generated from string database and visualized by Cytoscape (sorted by nodes degree and edges combined score). (c) PPI network was analyzed by the CytoHubb algorithms, and the top six targets in MCC score were identified as hub targets of SMYAD against AAA.

The 54 common targets were subjected to GO and KEGG pathway enrichment analysis to understand the potential mechanisms underlying the anti-AAA role of SMYAD. The BP results highlighted that SMYAD modulated a series of processes related to inflammatory response, defense response, apoptotic, cell migration and adhesion, and reactive oxygen species metabolic process. In addition, there were 572 GO entries of SMYAD against AAA, including 452 BP entries, 76 MF entries, and 44 CC entries. And the top seven *q*-value of each GO entry is shown in Supplementary Materials Figure S1: our experimental study has revealed that SMYAD could inhibit Bap/Ang II-induced inflammation. And the KEGG pathway analysis disclosed SMYAD might regulate a series of signalling pathways related to inflammation, such as TNF signalling pathway, NF-kappa B signalling pathway, and IL-17 signalling pathway,. More importantly, inflammatory responses were mediated by different classes of immune cells and mediums such as instance macrophage and interleukin, which participated and regulated in the above signalling pathway. These findings elucidated the possible mechanisms of SMYAD against AAA and suggested that SMYAD could be a potential drug for treating AAA. Finally, the related network diagram of SMYAD-Target-BP-KEGG-AAA was established and visualized by Cytoscape software in Figure 4(a).

### 3.8. PPI Network and Hub Targets

Integrated network analysis to explore the hub targets regulated by SMYAD against AAA. The common targets of SMYAD and AAA were subjected to PPI interaction network analysis. Although IL-6 and TNF were the low degree value nodes in the C-T network, they were the top two highest degree value targets in the PPI network (Figure 4(b)). According to CytoHubba algorithms, top six targets in MCC score, namely, IL-6, TNF, RELA, PTGS2, MMP9, and HSP90AA1, were reserved as the hub targets of SMYAD against AAA (Figure 4(c)).

### 3.9. Molecular Docking

Molecular docking algorithms execute quantitative predictions of binding ability, providing rankings of docked compounds-core targets based on the binding affinity. The targets, acquired from CytoHubba algorithms and literatures' supplement (NOS2, NOS3, ICAM1, PLA2G2A, SERPINE, and ESR1) [36–39], were regarded as core targets of SMYAD for treating AAA. And the corresponding proteins of these core targets were selected as receptors.

**Figure 5:**
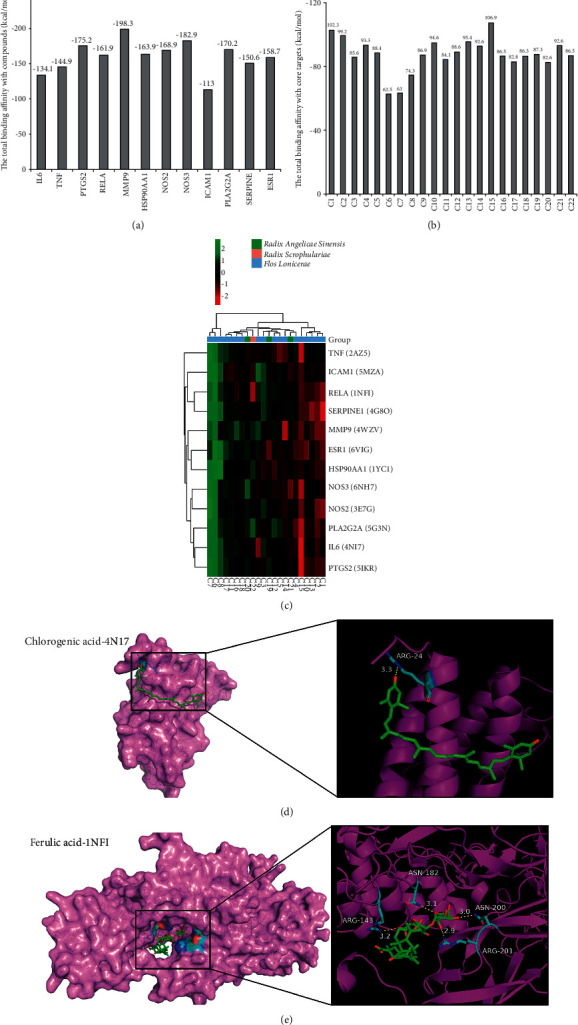
The molecular docking and sketch maps of active compounds binding to corresponding proteins of core targets. (a) The total binding energy of 12 core targets (receptors) and 22 compounds (ligands). (b) The transverse axis represented the 22 ligands; the longitudinal axis represented 12 receptors (with PBD ID). The color changed from green to red in the row represents the relative incensement of binding affinity; the combination of IL-6 and C15 had the more obvious difference in binding energy than other types. (c and d) The three-dimensional structure sites of the compounds and proteins are shown in the partial enlarged drawings. The protein backbone is represented as a cartoon. The compounds (carbon in green) and active site residues (carbon in blue) are shown in stick representation. The oxygen atom is shown as a red stick and hydrogen bonds are indicated as yellow dashed lines. (c) Site of chlorogenic acid with IL-6 (PDB ID 4NI7) and hydrogen bond formed between chlorogenic acid and 4NI7 of IL-6 on ARG-24 (3.3 Å). (d) Sites of ferulic acid with RELA (PDB ID 1NFI) and hydrogen bonds formed between ferulic acid and 1NFI protein of RELA on ARG-143 (3.2 Å), ARG-201 (2.9 Å), ASN-182 (3.1 Å), and ASN-200 (3.0 Å).

Similarly, C1-C22 compounds of SMYAD were chosen as ligands. A total of 264 times were docked, and the total binding energy of each receptor and ligand was (Figures 5(a) and 5(b)). According to the docking results, the compounds such as C15 (chlorogenic acid), C1 (luteolin), C2 (quercetin), C13 (cryptoxanthin), C10 (flavanone), C4 (kaempferol), C21 (harpagoside), and C14 (rhodoxanthin) play an important role in the treatment of AAA. Moreover, C15 had the lowest binding energy revealing that it probably be the most active compound in SMYAD; meanwhile, MMP9 had the lowest binding energy indicating that SMYAD was most likely to bind it and function as an AAA repressor. The differences of binding energy of each receptor with 22 ligands are shown in heat map (Figure 5(c)); the combination of IL-6 and C15 had the more obvious difference in binding energy than the other types. Furthermore, the docking process was performed to concretely describe the binding sites of SMYAD against AAA. Macrophage and IL-6 were verified to be involved in the development of AAA in our experimental study; in addition, RELA was implicated in the migration and infiltration of macrophages in AAA formation [40, 41]. Among the combinations of IL-6 and RELA with C1-C22, IL-6-C15 (chlorogenic acid) and RELA-C22 (ferulic acid) had the lowest binding energy. Thus, the above combinations were docked to explore the putative conformations. The most affinity binding conformation and the corresponding intermolecular interactions were identified. The results suggested that a hydrogen bond formed between chlorogenic acid and 4NI7 on ARG-246 (3.3 Å) (Figure 5(d)). Moreover, hydrogen bonds formed between ferulic acid and 1NFIonARG-143 (3.2 Å), ARG-201 (2.9 Å), ASN-182 (3.1 Å), and ASN-200 (3.0 Å) (Figure 5(e)).

## 4. Discussion

In the present study, SMYAD reduced the pathological dilation of abdominal aorta and the incidence of AAA in Bap-/Ang II-treated mice. Decreased AAA formation occurred concomitantly with a reduction of elastin fiber destruction, macrophage infiltration, and expression of IL-6. These above results clearly demonstrated that SMYAD alleviated over-activated inflammation and ameliorated the damage to abdominal aortic. Thus, SMYAD was a potentially protective agent for Bap/Ang II-induced AAA.

Smoking has long been considered a key risk factor for AAA. Bap, one of the major components of cigarettes, has been verified to contribute to AAA development. Furthermore, Bap could work synergistically with Ang II to induce AAA formation in mice by promoting macrophage infiltration and disruption of elastic lamella [35]. Bap/Ang II-induced damage to the abdominal aortic more accurate delineated the pathological mechanisms of AAA development.

An emerging concept is that AAA development is due to the inflammatory response, whereby many inflammatory cells and mediators have played important roles in regulating the activation of matrix-degrading proteins and smooth muscle cell apoptosis, resulting in the loss of medial elastic lamella and thinning of the tunica media [42, 43]. The pathological process of AAA starts with the infiltration and accumulation of inflammatory cells in the arterial wall [44, 45]. As the disease progresses, the inflammation in the arterial wall worsens [46]. Additionally, other inflammatory cells such as *T* and *B* cells are observed in AAA tissue samples and might play roles in AAA. Moreover, the migration and infiltration of macrophage may play a prominent role in the development of AAA [47–49]. Therefore, targeting macrophage-mediated vascular inflammation may be a potential treatment for the prevention of AAA. SMYAD has been verified to suppress the differentiation and activity of macrophage in mice [50]. Meanwhile, several studies have provided evidences that SMYAD contains multiple bioactivity effects, including anti-inflammatory, antioxidant, and regulation of platelet activation [51–54], which are closely related to the pathogenesis of AAA. Therefore, SMYAD was supposed to have intervention effects on AAA.

Our experimental study results suggested that SMYAD has a protective effect on the deterioration of AAA. We propose the pharmacological effects of SMYAD are as follows: Firstly, SMYAD contains anti-inflammatory factors. Besides quercetin, kaempferol, and beta-sitosterol, SMYAD also contains glycyrrhizin, which is well known for its anti-inflammatory activity [55]. These potent anti-inflammatory compounds could work synergistically to suppress macrophage infiltration, to alleviate inflammation-related destruction of elastin fiber. Then, SMYAD can decrease circulating levels of IL-6. As a main pro-inflammatory cytokine, IL-6 is a pleiotropic cytokine with roles in immunity and metabolism [56], and the excessive synthesis of IL-6 and dysregulation of IL-6 receptor signalling is involved in the pathological process of AAA [57]. AAA patients have significantly higher levels of serum IL-6 than either coronary heart disease patients or control subjects [58]. Signalling via IL-6 is a causal pathway in AAA [59, 60]. Recent studies have revealed that the blockade of IL-6 had a positive outcome on disease pathology, suggesting a novel strategy for therapeutic intervention [61–63]. In Bap-/Ang II-treated mice, elevated serum IL-6 was observed, and it was specifically inhibited by SMYAD, supporting the hypothesis that IL-6 plays an important role in AAA development in patients. In addition, there is a close relationship between IL-6 and macrophage in AAA [64]. These above results suggest that SMYAD suppressed macrophage activation and IL-6 expression and thus alleviated vascular inflammation and elastin lamella disruption during AAA development. Moreover, the results from the experiment were further validated in the next following bioinformatics prediction.

Next, the network pharmacology was performed to predict the active compounds, related pathways, and core targets involved in the SMYAD-provided protection on AAA. The enrichment results disclose the pivotal participation of inflammatory response, defense response, apoptotic, cell migration and adhesion, and reactive oxygen species metabolic in the SMYAD against AAA, as well as the collaborative involvement of the TNF signalling pathway, NF-kappa B signalling pathway, PI3K-Akt signalling pathway, and IL-17 signalling pathway [65–69]. Moreover, among those common targets obtained from PPI network, the first six hub targets with higher MCC were IL-6, TNF, RELA, PTGS2, MMP9, and HSP90AA1, and they all participate in regulating the processes of inflammatory reaction [70–73]. Therefore, inflammation and subsequent destruction of elastin lamella are crucially taken part in the development of AAA. Additionally, molecular docking further illuminated the effect of SMYAD against AAA lay in the good affinity and clear binding sites between the active compounds in SMYAD and the core targets in AAA formation. IL-6, as the most important core target in the PPI network and validated objective indicator in the experimental study, has better binding ability with chlorogenic acid than with other compounds. These findings point out the direction for the SMYAD purification and further study.

There are some limitations in our study: first, the intervention dosage of SMYAD was not reasonable enough, which leads to the effect of SMYAD high-dose group, was not better than that of the SMYAD-low-dose group. So, setting another drug such as statins may better reflect the effect of SMYAD. Second, we only test for the levels of IL-6 in circulating blood, not in the tissues of abdominal aortic, making it impossible to compare the differences of IL-6 in different tissues and get more information. Third, except for pathogenic target-IL-6, we do not know for sure whether there are protective targets in those core targets. Next, we intend to design a clinical study that focuses on the effect of SMYAD in AAA patients and screen for differential genes, to further clarify the mechanism of action.

## 5. Conclusions

Taken together, the experimental results and bioinformatics findings highlight the protective effect of SMYAD on AAA. Furthermore, SMYAD may be used in clinical to treat AAA, based on the identified pharmacological functions and clear signalling pathways. Moreover, the potent compounds of SMYAD against AAA were identified, supporting it as an effective medicine with clear targets for action. Thus, it may be a safe and promising drug candidate for AAA.

## Figures and Tables

**Table 1:** The major active compounds of Si-Miao-Yong-An decoction.

Compound number	Name	PubChem CID	Molecular formula	MW
*Flos Lonicerae (Jin-Yin-Hua)*				
C1	Luteolin	5280445	C_15_H_10_O_6_	286.24
C2	Quercetin	5280343	C_15_H_10_O_7_	302.23
C3	Beta-sitosterol	86821	C_29_H_50_O	414.7
C4	Kaempferol	5280863	C_15_H_10_O_6_	286.24
C5	Stigmasterol	5280794	C_29_H_48_O	412.7
C6	Mandenol	5282184	C_20_H_36_O_2_	308.5
C7	Ethyl linoleate	11001	C_20_H_36_O_2_	308.5
C8	Phytofluenes	94171	C_40_H_62_	542.9
C9	Beta-carotene	573	C_40_H_56_	536.9
C10	Flavanone	10251	C_15_H_12_O_2_	224.25
C11	8-Indolizine carboxylic acid	11106605	C_11_H_15_NO_3_	209.24
C12	Chrysoeriol	5280666	C_16_H_12_O_6_	300.26
C13	Cryptoxanthin	182237	C_40_H_56_O	552.9
C14	Rhodoxanthin	92741	C_40_H_50_O_2_	562.8
C15	Chlorogenic acid	1794427	C_16_H_18_O_9_	354.3
C16	Epi-Vogeloside	14192588	C_17_H_24_O_10_	388.4
C17	Xylostosidine	4333465	C_18_H_25_NO_8_S	415.5
C18	Corymbosin	10970376	C_19_H_18_O_7_	358.3

*Radix Scrophulariae (Xuan-Shen)*				
C3	Beta-sitostero	86821	C_29_H_50_O	414.7
C19	Methyl benzoate	11973336	C_17_H_18_O_6_	318.32
C20	Sugiol	275529	C_20_H_28_O_2_	300.4
C21	Harpagoside	5281542	C_24_H_30_O_11_	494.5

*Radix Angelicae Sinensis (Dang-Gui)*				
C3	Beta-sitosterol	86821	C_29_H_50_O	414.7
C5	Stigmasterol	5280794	C_29_H_48_O	412.7
C22	Ferulic acid	445858	C_10_H_10_O_4_	194.2

Seventy-eight compounds of *Radix Glycyrrhizin* (Gan-Cao) are shown in Supplementary File 2.

## Data Availability

The datasets used during the present study are available from the corresponding author upon reasonable request.

## Supplementary Materials

Supplementary Materials Figure S1: the top seven *q*-values of biological process (BP) entries, molecular function (MF) entries, and cell component (CC) entries related to Si-Miao-Yong-An decoction (SMYAD) against abdominal aortic aneurysm (AAA). Table S1: the body and heart weights of mice in the experimental study. Table S2: the active compounds of *Radix Glycyrrhizin*. Figure S2: the top seven *q*-values of biological process (BP) entries, molecular function (MF) entries, and cell component (CC) entries.
