# Supplementary material for: Exploring the Effect and Mechanism of Si-Miao-Yong-An Decoction on Abdominal Aortic Aneurysm Based on Mice Experiment and Bioinformatics Analysis
Source: Evid Based Complement Alternat Med. 2022 May 31;2022:4766987. doi: 10.1155/2022/4766987 (PMC9173986; doi:10.1155/2022/4766987)

## Supplementary Materials

File 1: The body and heart weights of mice in experimental study.

**Table 1S** The body and heart weights of mice at Day1 and Day 43

| Group | Body weight (g, ‾x± SD)  Day 1 Day 43 | | Heart weight (g, ‾x± SD)  Day 43 |
| --- | --- | --- | --- |
| Control | 29.75±1.13^■^ | 29.49±1.62^◆^ | 0.40±0.08^☆^ |
| Bap+Ang II | 29.72±1.17^■^ | 28.84±1.46^◆^ | 0.43±0.07^☆^ |
| Bap+Ang II+SL | 29.65±1.12^■^ | 29.11±1.49^◆^ | 0.42±0.06^☆^ |
| Bap+Ang II+SH | 29.52±1.19^■^ | 28.99±1.39^◆^ | 0.41±0.09^☆^ |

■,◆,☆ *P* > 0.05, respectively.

File 2: The active compounds of *Radix Glycyrrhizin*.

**Table 2S** The activer compounds of *Radix Glycyrrhizae*

| Compound number | Name | | PubChem CID | | | Molecular formula | | MW |
| --- | --- | --- | --- | --- | --- | --- | --- | --- |
| *Radix Glycyrrhizae* (Gancao) | | | | | | | | |
| C2  C3  C4  C23  C24  C25  C26  C27  C28  C29  C30  C31  C32  C33  C34  C35  C36  C37  C38  C39  C40  C41  C42  C43  C44  C45  C46  C47  C48  C49  C50  C51  C52  C53  C54  C55  C56  C57  C58  C59  C60  C61  C62  C63  C64  C65  C66  C67  C68  C69  C70  C71  C72  C73  C74  C75  C76  C77  C78  C79  C80  C81  C82  C83  C84  C85  C86  C87  C88  C89  C90  C91  C92  C93  C94  C95  C96  C97 | | Quercetin  Beta-sitosterol  Kaempferol  Glycyrrhizin  Betulic acid  Kumatakenin  Isorhamnetin  Formononetin  Calycosin  Licochalcone  Vestitol  Maackiain  DL-Liquiritigenin  Glycyrol  Medicarpin  Lupiwighteone  Naringenin  Shinflavanone  Glyasperin B  Glyasperin F  Glyasperin C  Isotrifoliol  Kanzonol B  Kanzonol W  Licofuranocoumarin  Semilicoisoflavone B  Glepidotin A  Glepidotin B  Phaseolinisoflavan  Glypallichalcone  Kanzonol U  Licochalcone B  Licochalcone G  Licoarylcoumarin  Licoricone  Gancaonin A  Gancaonin B  Gancaonin L  Gancaonin M  Glycyrin  Licocoumarone  Edudiol  Licoisoflavone B  Licoisoflavanone  Shinpterocarpin  5-Prenylbutein  Licopyranocoumarin  5-Carboxylate  Glyzaglabrin  (R)-Glabridin  Glabranine  Glabrene  Glabrone  Hedysarim B  Heptaen-9-one  Eurycarpin A  Medicocarpin  5'-Prenyleriodictyol  Isobavachin  Isoglycyrol  Isolicoflavonol  Isoformononetin  1-Methoxyphaseollidin  3,3'-Dimethoxyquercetin  4'-Methoxyglabridin  3'-Methoxyglabridin  4'-O-Methylglabridin  7-Hydroxy-Coumarin  Kanzonol F  3-Phenylcoumarin  2-Methylisoflavone  Gancaonin G  Gancaonin H  Glycyrrhiza flavonol A  Licoagroisoflavone  Odoratin  Xambioona  Dehydroglyasperin C | | 5280343  86821  5280863  3495  2371  5318869  5281654  5280378  5280448  3923  92503  363863  1889  5320083  623060  5317480  932  10340350  480784  392442  45783134  5318679  10881804  15380912  5319001  5481948  5281619  3512635  4484952  127029  10542808  480788  49856081  10090416  5319013  5317478  5317479  14604077  14604078  480787  503731  44257469  5481234  392443  85114192  72776640  122851  5317769  5317777  4484219  3144815  480774  5317652  11558452  11602329  5317300  44257429  11725803  11609510  124050  5318585  3764  44257468  5316900  10338211  5319439  5319664  5318437  131752864  25015742  268208  480780  5481949  5317765  12108704  13965473  14769500  480775 | [C_15_H_10_O_7_](https://pubchem.ncbi.nlm.nih.gov/#query=C15H10O7)  C_29_H_50_O  [C_15_H_10_O_6_](https://pubchem.ncbi.nlm.nih.gov/#query=C15H10O6)  C_42_H_62_O_16_  [C_30_H_48_O_3_](https://pubchem.ncbi.nlm.nih.gov/#query=C30H48O3)  [C_17_H_14_O_6_](https://pubchem.ncbi.nlm.nih.gov/#query=C17H14O6)  [C_16_H_12_O_7_](https://pubchem.ncbi.nlm.nih.gov/#query=C16H12O7)  [C_16_H_12_O_4_](https://pubchem.ncbi.nlm.nih.gov/#query=C16H12O4)  [C_16_H_12_O_5_](https://pubchem.ncbi.nlm.nih.gov/#query=C16H12O5)  [C_21_H_22_O_4_](https://pubchem.ncbi.nlm.nih.gov/#query=C21H22O4)  [C_16_H_16_O_4_](https://pubchem.ncbi.nlm.nih.gov/#query=C16H16O4)  [C_16_H_12_O_5_](https://pubchem.ncbi.nlm.nih.gov/#query=C16H12O5)  [C_15_H_12_O_4_](https://pubchem.ncbi.nlm.nih.gov/#query=C15H12O4)  [C_21_H_18_O_6_](https://pubchem.ncbi.nlm.nih.gov/#query=C21H18O6)  [C_16_H_14_O_4_](https://pubchem.ncbi.nlm.nih.gov/#query=C16H14O4)  [C_20_H_18_O_5_](https://pubchem.ncbi.nlm.nih.gov/#query=C20H18O5)  [C_15_H_12_O_5_](https://pubchem.ncbi.nlm.nih.gov/#query=C15H12O5)  [C_25_H_26_O_4_](https://pubchem.ncbi.nlm.nih.gov/#query=C25H26O4)  [C_21_H_22_O_6_](https://pubchem.ncbi.nlm.nih.gov/#query=C21H22O6)  [C_20_H_18_O_6_](https://pubchem.ncbi.nlm.nih.gov/#query=C20H18O6)  [C_21_H_24_O_5_](https://pubchem.ncbi.nlm.nih.gov/#query=C21H24O5)  [C_16_H_10_O_6_](https://pubchem.ncbi.nlm.nih.gov/#query=C16H10O6)  [C_20_H_18_O_4_](https://pubchem.ncbi.nlm.nih.gov/#query=C20H18O4)  [C_20_H_16_O_5_](https://pubchem.ncbi.nlm.nih.gov/#query=C20H16O5)  [C_21_H_20_O_7_](https://pubchem.ncbi.nlm.nih.gov/#query=C21H20O7)  [C_20_H_16_O_6_](https://pubchem.ncbi.nlm.nih.gov/#query=C20H16O6)  [C_20_H_18_O_5_](https://pubchem.ncbi.nlm.nih.gov/#query=C20H18O5)  [C_20_H_20_O_5_](https://pubchem.ncbi.nlm.nih.gov/#query=C20H20O5)  [C_20_H_20_O_4_](https://pubchem.ncbi.nlm.nih.gov/#query=C20H20O4)  [C_17_H_16_O_4_](https://pubchem.ncbi.nlm.nih.gov/#query=C17H16O4)  [C_19_H_16_O_4_](https://pubchem.ncbi.nlm.nih.gov/#query=C19H16O4)  [C_16_H_14_O_5_](https://pubchem.ncbi.nlm.nih.gov/#query=C16H14O5)  [C_21_H_22_O_5_](https://pubchem.ncbi.nlm.nih.gov/#query=C21H22O5)  [C_21_H_20_O_6_](https://pubchem.ncbi.nlm.nih.gov/#query=C21H20O6)  [C_22_H_22_O_6_](https://pubchem.ncbi.nlm.nih.gov/#query=C22H22O6)  [C_21_H_20_O_5_](https://pubchem.ncbi.nlm.nih.gov/#query=C21H20O5)  [C_21_H_20_O_6_](https://pubchem.ncbi.nlm.nih.gov/#query=C21H20O6)  [C_20_H_18_O_6_](https://pubchem.ncbi.nlm.nih.gov/#query=C20H18O6)  [C_21_H_20_O_5_](https://pubchem.ncbi.nlm.nih.gov/#query=C21H20O5)  [C_22_H_22_O_6_](https://pubchem.ncbi.nlm.nih.gov/#query=C22H22O6)  [C_20_H_20_O_5_](https://pubchem.ncbi.nlm.nih.gov/#query=C20H20O5)  [C_21_H_22_O_5_](https://pubchem.ncbi.nlm.nih.gov/#query=C21H22O5)  [C_20_H_16_O_6_](https://pubchem.ncbi.nlm.nih.gov/#query=C20H16O6)  [C_20_H_18_O_6_](https://pubchem.ncbi.nlm.nih.gov/#query=C20H18O6)  [C_20_H_18_O_4_](https://pubchem.ncbi.nlm.nih.gov/#query=C20H18O4)  [C_20_H_20_O_5_](https://pubchem.ncbi.nlm.nih.gov/#query=C20H20O5)  [C_21_H_20_O_7_](https://pubchem.ncbi.nlm.nih.gov/#query=C21H20O7)  [C_31_H_44_O_6_](https://pubchem.ncbi.nlm.nih.gov/#query=C31H44O6)  [C_16_H_10_O_6_](https://pubchem.ncbi.nlm.nih.gov/#query=C16H10O6)  [C_20_H_20_O_4_](https://pubchem.ncbi.nlm.nih.gov/#query=C20H20O4)  [C_20_H_20_O_4_](https://pubchem.ncbi.nlm.nih.gov/#query=C20H20O4)  [C_20_H_18_O_4_](https://pubchem.ncbi.nlm.nih.gov/#query=C20H18O4)  [C_20_H_16_O_5_](https://pubchem.ncbi.nlm.nih.gov/#query=C20H16O5)  [C_16_H_10_O_6_](https://pubchem.ncbi.nlm.nih.gov/#query=C16H10O6)  [C_17_H_12_O_7_](https://pubchem.ncbi.nlm.nih.gov/#query=C17H12O7)  [C_20_H_18_O_5_](https://pubchem.ncbi.nlm.nih.gov/#query=C20H18O5)  [C_22_H_24_O_9_](https://pubchem.ncbi.nlm.nih.gov/#query=C22H24O9)  [C_20_H_20_O_6_](https://pubchem.ncbi.nlm.nih.gov/#query=C20H20O6)  [C_20_H_20_O_4_](https://pubchem.ncbi.nlm.nih.gov/#query=C20H20O4)  [C_21_H_18_O_6_](https://pubchem.ncbi.nlm.nih.gov/#query=C21H18O6)  [C_20_H_18_O_6_](https://pubchem.ncbi.nlm.nih.gov/#query=C20H18O6)  [C_16_H_12_O_4_](https://pubchem.ncbi.nlm.nih.gov/#query=C16H12O4)  [C_21_H_22_O_5_](https://pubchem.ncbi.nlm.nih.gov/#query=C21H22O5)  [C_17_H_14_O_7_](https://pubchem.ncbi.nlm.nih.gov/#query=C17H14O7)  [C_21_H_22_O_5_](https://pubchem.ncbi.nlm.nih.gov/#query=C21H22O5)  [C_21_H_22_O_5_](https://pubchem.ncbi.nlm.nih.gov/#query=C21H22O5)  [C_21_H_22_O_4_](https://pubchem.ncbi.nlm.nih.gov/#query=C21H22O4)  [C_20_H_18_O_4_](https://pubchem.ncbi.nlm.nih.gov/#query=C20H18O4)  [C_26_H_28_O_5_](https://pubchem.ncbi.nlm.nih.gov/#query=C26H28O5)  [C_16_H_12_O_6_](https://pubchem.ncbi.nlm.nih.gov/#query=C16H12O6)  [C_18_H_14_O_4_](https://pubchem.ncbi.nlm.nih.gov/#query=C18H14O4)  [C_21_H_20_O_5_](https://pubchem.ncbi.nlm.nih.gov/#query=C21H20O5)  [C_25_H_24_O_6_](https://pubchem.ncbi.nlm.nih.gov/#query=C25H24O6)  [C_20_H_18_O_7_](https://pubchem.ncbi.nlm.nih.gov/#query=C20H18O7)  [C_20_H_16_O_5_](https://pubchem.ncbi.nlm.nih.gov/#query=C20H16O5)  [C_17_H_14_O_6_](https://pubchem.ncbi.nlm.nih.gov/#query=C17H14O6)  [C_25_H_24_O_4_](https://pubchem.ncbi.nlm.nih.gov/#query=C25H24O4)  [C_21_H_22_O_5_](https://pubchem.ncbi.nlm.nih.gov/#query=C21H22O5) | | 302.23  414.7  286.24  822.9  456.7  314.29  316.26  268.26  284.26  338.4  272.29  284.26  256.25  366.4  270.28  338.4  272.25  390.5  370.4  354.4  356.4  298.25  322.4  336.3  384.4  352.3  338.4  340.4  324.4  284.31  308.3  286.28  354.4  368.4  382.4  352.4  368.4  354.4  352.4  382.4  340.4  354.4  352.3  354.4  322.4  340.4  384.4  512.7  298.25  324.4  324.4  322.4  336.3  298.25  328.27  338.4  432.4  356.4  324.4  366.4  354.4  268.26  354.4  330.29  354.4  354.4  338.4  322.4  420.5  300.26  294.3  352.4  420.5  370.4  336.3  314.29  388.5  354.4 | |

File 3: The top seven *q*-values of biological process (BP) entries, molecular function (MF) entries and cell component (CC) entries related to Si-Miao-Yong-An decoction (SMYAD) against abdominal aortic aneurysm (AAA).


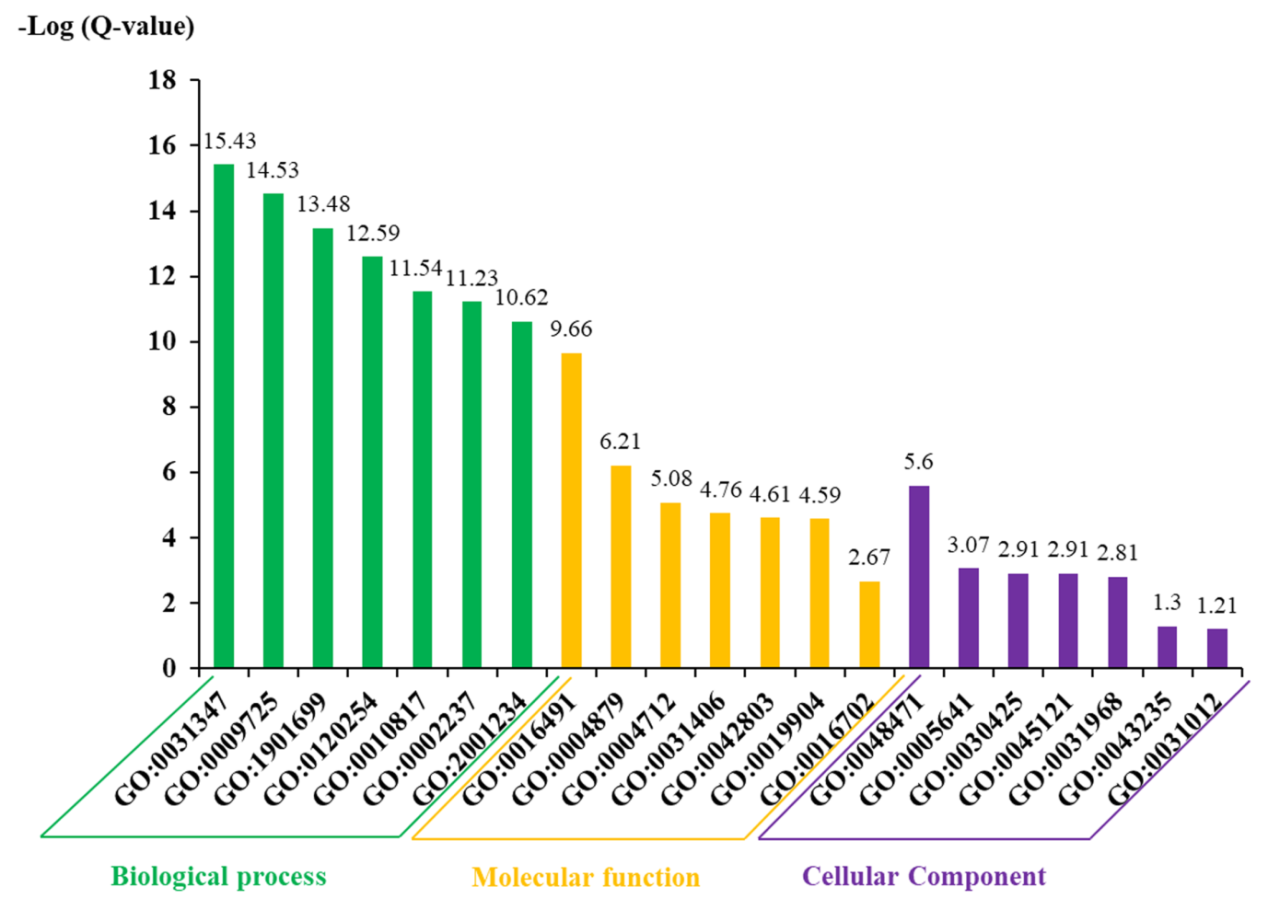

Supplement: Supplementary Materials — Figure S1: the top seven q-values of biological process (BP) entries, molecular function (MF) entries, and cell component (CC) entries related to Si-Miao-Yong-An decoction (SMYAD) against abdominal aortic aneurysm (AAA). Table S1: the body and heart weights of mice in the experimental study. Table S2: the active compounds of Radix Glycyrrhizin. Figure S2: the top seven q-values of biological process (BP) entries, molecular function (MF) entries, and cell component (CC) entries. [file 4766987.f1.docx]
